# Prognostic value of combined preoperative phase angle and handgrip strength in cardiac surgery

**DOI:** 10.1186/s13019-022-01970-z

**Published:** 2022-09-03

**Authors:** Mairi Panagidi, Αndreas S. Papazoglou, Dimitrios V. Moysidis, Elpiniki Vlachopoulou, Marios Papadakis, Evangelia Kouidi, Antonios Galanos, Georgios Tagarakis, Kyriakos Anastasiadis

**Affiliations:** 1 Department of Cardiothoracic Surgery, AHEPA University Hospitalof Thessaloniki, Thessaloniki, Greece; 2 First Department of Cardiology, AHEPA University Hospital of Thessaloniki, Thessaloniki, Greece; 3 Department of Nutritional Sciences, International Hellenic University, Thessaloniki, Greece; 4 University Witten-Herdecke, Witten, Germany; 5 Laboratory of Sports Medicine, Department of Physical Education and Sports Sciences, Aristotle University of Thessaloniki, Thessaloniki, Greece; 6 Department of Medicine, National and Kapodistrian University of Athens, Athens, Greece

**Keywords:** Cardiac surgery, Phase angle, Handgrip strength, Coronary artery bypass grafting, All-cause mortality

## Abstract

**Objectives:**

Phase angle (PA) constitutes a bioelectrical impedance measurement, indicating cell membrane health and integrity, hydration, and nutritional status. Handgrip strength (HS) has been also associated with body composition, nutritional status, inflammation, and functional ability in several chronic diseases. Although their prognostic significance as independent biomarkers has been already investigated regarding the outcomes of a cardiac surgery, our study is the first one to assess the combined predictive value of preoperative PA and HS.

**Design and methods:**

HS and PA measurements were performed preoperativelyin 195 patients undergoing cardiac surgery. The association ofthe combination of HS and PAwith all-cause mortality rates was the primary study outcome, while its association with the intensive care unit (ICU) length of stay (LOS) was the secondary one.

**Results:**

PA was positively correlated with HS (r = 0.446, *p* < 0.005) and negatively with EuroSCORE II (r = − 0.306 *p* < 0.005). The combination of PA < 5.15 and HS < 25.5 was associated with higher one-year all-cause mortality (OR = 9.28; 95% CI 2.50–34.45; *p* = 0.001) compared to patients with PA > 5.15 and HS > 25.5, respectively. Patients with combined lower values of PA and HS (PA < 5.15 and HS < 30.7) were at higher risk of prolonged ICU LOS (OR = 4.02; 95% CI 1.53–10.56; *p* = 0.005) compared to those with higher PA–HS (PA > 5.15–HS > 30.7). The combination of PA–HS was also significantly linked with EuroSCORE II.

**Conclusion:**

The combination of low preoperative PA and HS values was significantly associated with higher risk of all-cause mortality at 12 months and prolonged ICU LOS; thereby it might serve as a clinically useful prognostic biomarker after cardiac surgery procedures.

**Supplementary Information:**

The online version contains supplementary material available at 10.1186/s13019-022-01970-z.

## Introduction

Predicting post-procedural morbidity and mortality remains challenging despite the development of many scoring systems and prognostic algorithms in cardiac surgery [1, 2]. Hence, the assessment of novel biomarkers capable of guiding the modern physician in choosing the optimal, individualized, treatment for patients based on clinical prediction is of outmost importance [3].

The latest international guidelines support a more individualized patient management based on group discussion among cardiac specialists (i.e. “heart team”) and on the utilization of novel biomarkers such as frailty tests [4].Indicators of cellular integrity, functional capacity, and biological vulnerability have been recently proposed as preoperative risk factors associated with short- and long-term patient outcomes [5].In this context, phase angle (PA) [6] assessed with bioelectric impedance and handgrip strength (HS) [7] measured using a hand dynamometer emerge as valuable tools of promising cost-effectiveness with high reliability and accuracy, without posing a significant burden to the examiner. More specifically, PA, reflecting the resistance and reactance of cell membranes, and HS, reflecting the physical performance, have been already associated with sarcopenia, systemic inflammation, and increased morbidity and mortality burden [8].

In this prospective study enrolling patients undergoing selective open-heart surgery, we examined whether the combination of PA and HS values was associated with higher rates of one-year mortality, early morbidity and higher intensive care unit (ICU) stay. Our aim was to investigate the role of PA and HS in predicting clinical outcomes and enhancing pre-operative risk stratification in cardiac surgery patients.

## Materials and methods

### Study population and eligibility criteria

The study population comprised adult patients undergoing scheduled selective coronary artery bypass grafting (CABG) surgery, individual valve replacement or repair, or any combination of these procedures at the Cardio-Thoracic Surgery Department of AHEPA University Hospital between December 2018 and October 2019.

The exclusion criteria were: i. age < 18 years, ii. hemodynamic instability requiring urgent surgery, iii. urgent surgery for aortic dissection, iv. any major adverse intra-operative outcome, v. congenital heart disease, vi. recent cardiac surgery during the prior three months, and vii. presence of any implantable device. The study protocol has been approved by the Scientific Board of AHEPA University Hospital as well as by Ethics Committee of the Aristotle University of Thessaloniki. Written informed consent was obtained pre-operatively from every participant.

### Data extraction

On admission, demographic, anthropometric and clinical data [age, gender, and body mass index (BMI), type of surgery, EuroSCORE II, left ventricular ejection fraction (LVEF) and comorbidities] were recorded for each individual. Hospitalization data such as cardiopulmonary bypass (CPB) time, duration of mechanical ventilation, occurrence of post-operative complications, length of stay in the Intensive Care Unit (ICU) and postoperative length of hospital stay were also noted.

PA was measured using bioelectrical impedance method on the first pre-operative day by a blinded researcher trained in this technique. A simple quadrupole measurement was applied to the right side of the body using four-surface electrodes (QuadScan 4000, Bodystat, Isle of White, UK). Thereby, resistance (restriction of current flow) and reactance (capacitance of cell membranes to block current) were measured. The primary 50 kHz resistance and reactance data were used to calculate PA (tangent of reactance / resistance X 180°, divided by p and expressed in degrees).

HS was also assessed preoperatively using a portable hydraulic dynamometer (Takei 5001 GripA, Takei Scientific Instruments CO, Japan). The HS test was performed in the sitting position, having their elbow flexed at 90°, whilst pressing the dynamometer with the dominant hand at full force for three seconds. After three repetitions of the test with an interval of one minute to avoid fatigue, the best performance was recorded in kilograms (kg).

### Outcomes of interest

Primary study outcome was deemed all-cause mortality, as assessed at last available telephonic follow-up or as determined by electronic medical health records. The length of ICU stay was considered as the secondary study outcome.

### Statistical analysis

Continuous variables are presented with mean and standard deviation (SD) or with median and intra-quartile range (IQR) depending on data normality. Categorical variables are presented with frequencies (n) and percentages (%). Regularity of data distribution was checked using the Kolmogorov–Smirnov test.

The correlation of PA and HS with outcomes of interest was performed via t-test or Mann Whitney test for independent samples. Logistic regression was performed to detect the independent effect of demographic and clinical indicators on the outcomes of interest. The predictive value of PA and HS was evaluated through receiver operator characteristics (ROC) analyses and calculated Areas Under the Curve (AUC). Cut-off points of PA and HS that maximize sensitivity and specificity for risk stratification were evaluated via calculating Positive Predictive Value (PPV) and Negative Predictive Value (NPV). Due to the non-standardization of PA and HS values, statistical tests of collinearity were executed for each one of the performed multivariate analyses to investigate if there is any source of collinearity which could decrease the value of the independent effect. All statistical analyses were performed with the statistical package SPSS version 21 (IBM Corporation, Somers, NY, USA). The p-value of less than 0.05 was defined as the level of statistical significance.

## Results

Our study included 195 patients with a mean age of 67 years (SD: 9 years) of whom 150 were men (76.9%) and 45 women (23.1%). Of included patients, 90 suffered from coronary artery disease (46.2%), 87 (44.6%) from valvular disease and 18 from both (9.2%). Median EuroSCORE II was equal to 2.6%(0.6–15.5). Demographic and clinical characteristics of the overall study population and of the patients who survived compared to those who died during follow-up, are presented in Tables 1 and 2, respectively. PA was positively correlated with HS (r = 0.446 *p* < 0.005) and negatively with EUROSCORE II (r = − 0.306 *p* < 0.005) (Table 3). All patients were followed up for a median period of 1 year and no patients were lost during follow-up (drop-out rate = 0%). The median hospital and ICU stay were 9 days (IQR: 3 days) and 2 days (IQR: 2 days), respectively.

**Table 1:** Demographic and clinical characteristics of the overall study population

Age (years)	67.18 ± 9.14
Male gender (%)	150(76.9)
Body Mass Index (kg/m^2^)	27.75 ± 4.34(18.1–39.6)
Cardiopulmonary bypass time (minutes)	114.5(23–290)
Coronary Artery Disease/Valvular disease / both (%)	90(46.2)/87(44.6)/18(9.2)
Type II diabetes mellitus (%)	89(45.6)
Chronic kidney disease (%)	66(33.8) 9
Euroscore II (units)	2.6 (0.6–15.5)
Phase angle (^o^)	5.52 ± 1.47(2.7–16.0)
Handgrip strength (calf circumference)	27.48 ± 9.16(5–51)
Postoperative infections (%)	22(11.3)
Intensive Care Unit stay over 1 day (%)	99(50.8)
Mechanical ventilation over 1 day (%)	51(26.2)
In-hospital postoperative stay more than 7 days (%)	147(75.4)
Postoperative complications (reopening, pulmonary embolism, peripheral thrombosis, septic condition, in-hospital mortality) (%)	51(26.2)
All-cause mortality (%)	27(13.8)

Continuous variables are recorded as mean ± standard deviation or median (interquartile range), while categorical ones as n (%)

**Table 2:** Comparison of demographic and clinical characteristics between patients who survived and patients who died during follow-up

	Dead patients (n = 27)	Alive patients (n = 168)	*p* value (dead vs alive)
Age (years)	69.2 ± 7.6	66.9 ± 9.3	0.08
Male gender	23 (85%)	127 (76%)	0.37
Body Mass Index (kg/m2)	27.8 ± 4.8	27.8 ± 4.4	0.82
Cardiopulmonary bypass time (minutes)	135 ± 53.8	118 ± 44.2	0.17
Type of surgery:			0.45
Coronary Artery disease	11 (41%)	79 (47%)	
Valvular disease	12 (44%)	75 (45%)	
Combined	4 (15%)	14 (8%)	
Type II diabetes mellitus	12 (44%)	77 (46%)	0.84
Chronic kidney disease	9 (33%)	57 (34%)	0.48
Euroscore II (units)	3.8 (2.1)	2.4 (1.8)	< 0.01
Phase angle (units)	5 ± 1.4	5.6 ± 1.5	0.04
Handgrip strength (units)	22.6 ± 8.4	28.1 ± 9.1	< 0.01
Post-operative infections	12 (44%)	10 (6%)	< 0.01
Intensive Care Unit stay over 1 day	22 (81%)	77 (46%)	< 0.01
Mechanical ventilation over 1 day	20 (74%)	31 (18%)	< 0.01
In-hospital post-operative stay more than 7 days	21 (78%)	126 (75%)	0.20

Continuous variables are recorded as mean (± SD) or median (IQR)

**Table 3:** Correlation of the PA indicator with HS and EuroScore II

Correlation Indicators		PA		
		Correlation Coefficient	*p* value	N
Spearman ‘s rho	HS	0.446	** < 0.005**	195
	EUROSCORE II(%)	− 0.306	** < 0.005**	195

Bold values represent statistically significant values (*p* < 0.05)

Regarding the primary study outcome, 27 patients (13.8%) died from any cause after one-year follow-up. PA, HS and their combination had a significant yet fair predictive value for all-cause mortality; PA: AUC (95% CI) 0.657 (0.54–0.77); *p* = 0.009, HS: AUC (95% CI) 0.659 (0.5–0.78); *p* = 0.008, and their combination: AUC (95% CI) 0.671 (0.56–0.78); *p* = 0.004 (Table 4).

**Table 4:** ROC analysis of the PA and HS indicators and their combination in relation to mortality and ICU stay

Predictors	Variable	AUC(95%CI)*	*p *value	Cut-off point	Sensitivity	Specificity	PPV*	NPV*
PA^†^	Mortality ICU LOS	**0.657** (0.540.77) **0.600** (0.52–0.68)	0.009 0.016	5.15 5.15	67% 56%	61% 70%	21% 65%	92% 60%
HS^†^	Mortality ICU LOS ICU LOS	**0.659** (0.55–0.78) **0.586** (0.51–0.67)	0.008 0.040	25.5 30.75	67% 71%	63% 47%	22% 57%	92% 60%
Combina-tion^†^^PA/HS^	Mortality ICU LOS	**0.671** (0.56–0.78) **0.597** (0.52–0.68)	0.004 0.019	**5.15/25.5** **5.15/30.75**	67% 63%	62% 59%	22% 61%	92% 60%

Bold values represent statistically significant values (*p* < 0.05)

^†^Lower values ​​indicate a poor outcome

^*^AUC: Area Under the Curve; CI: confidence interval; PPV: positive prognostic value; NPV: negative prognostic value

The PA–HS combination had a significant effect on mortality occurrence [*p* = 0.009]. Patients with PA < 5.15 and HS < 25.5 were 5 times more likely to die [5.13 (1.84–14.27); *p* = 0.002] when compared to those with PA > 5.15–HS > 25.5 (Table 5). The multivariate analysis, presented in Table 6, yielded that female gender [0.27 (0.08–0.94); *p* = 0.040], Euroscore II [1.59 (1.17–2.14); *p* = 0.003] and the combination of PA–HS [*p* = 0.005] had an independent effect on mortality. Regarding the PA–HS combination, patients with PA < 5.15 and HS < 25.5 were 9.3 times more likely to die [9.28 (2.50–34.45); *p* = 0.001] in relation to those with PA > 5.15–HS > 25.5 (Table 6).

**Table 5:** Univariate logistic regression of all-cause mortality with the combination of PA and HS

	OR	95% CI		*p* value
Combination PA–HS				0.009
PA > 5.15–HS > 25.5	1.00 (reference)			–
PA > 5.15–HS < 25.5	1.62	0.38	6.99	0.514
PA < 5.15–HS > 25.5	1.39	0.33	5.95	0.655
PA < 5.15–HS < 25.5	5.13	1.84	14.27	0.002

**Table 6:** Multivariate logarithmic regression of all-cause mortality with the combination of PA and HS adjusted to clinical indicators

Variables	OR	95% CI		*p* value
Female Gender	0.27	0.08	0.94	0.040
Age	1.00	0.94	1.05	0.869
Diabetes ΙΙ	0.85	0.31	2.30	0.746
Chronic Kidney Disease	0.53	0.19	1.50	0.233
EuroscoreΙΙ	1.59	1.17	2.14	0.003
Ejection Fraction	0.96	0.91	1.02	0.226
Combination PA–HS				0.005
PA > 5.15–HS > 25.5	1.00 (reference)			–
PA > 5.15–HS < 25.5	2.97	0.57	15.41	0.195
PA < 5.15–HS > 25.5	1.65	0.34	8.02	0.533
PA < 5.15–HS < 25.5	9.28	2.50	34.45	0.001

Α multivariate regression model for the prediction of 1-year mortality using PA and HS as continuous variables was also performed and is presented in Additional file 1: Table S1. According to this analysis, increased HS was independently linked with decreased all-cause mortality rates (aOR = 0.90, 95% CI: 0.84–0.96).

With regard to the secondary study outcome, the PA–HS combination was significantly associated with prolonged stay in the ICU [*p* = 0.002]. Patients with PA < 5.15 and HS < 30.7 were 4 times more likely to stay in the ICU for more than 1 day [4.14 (1.95–8.80); *p* = 0.001] in comparison with those having PA > 5.15 and HS > 30.7 (Table 7). PA, HS and their combination had also a significant yet poor predictive value for the prolonged stay in the ICU; PA: AUC (95% CI) = 0.600 (0.52–0.68); *p* = 0.016, HS: AUC (95% CI) = 0.586 (0.51–0.67); *p* = 0.040, and their combination: AUC (95% CI) = 0.597 (0.52–0.68); *p* = 0.019 (Table 4).

**Table 7:** Univariate logistic regression of prolonged stay in the ICU with the combination of PA and HS

	OR	95%CI		*p* value
Combination PA–HS				0.002
PA > 5.15–HS > 30.7	1.00 (reference)			–
PA > 5.15–HS < 30.7	2.01	0.93	4.36	0.076
PA < 5.15–HS > 30.7	3.97	1.27	12.43	0.018
PA < 5.15–HS < 30.7	4.14	1.95	8.80	< 0.005

Multivariate analysis for the prediction of prolonged ICU stay demonstrated that female gender [0.35 (0.15–0.83); *p* = 0.017], EuroSCORE II [1.71 (1.27–2.30); *p* < 0.005], left ventricular ejection fraction [1.05 (1.01–1.10); *p* = 0.018] and the combination of PA–HS [*p* = 0.038] were independent predictors. Regarding the combination of those indicators, patients with PA < 5.15 and HS < 30.7 were found to be 4 times more likely to stay in the ICU for more than 1 day [4.02 (1.53–10.56); *p* = 0.005] compared to those with PA > 5.15–HS > 30.7 (Table 8).

**Table 8:** Multivariate logistic regression of prolonged stay in the ICU with the combination of PA–HS adjusted to clinical indicators

Variables	OR	95% CI		*p* value
Female gender	0.35	0.15	0.83	0.017
Age	1.03	0.99	1.07	0.152
Diabetes ΙΙ	1.57	0.81	3.06	0.182
Chronic Kidney Disease	0.54	0.26	1.12	0.096
Euroscore ΙΙ	1.71	1.27	2.30	< 0.005
Ejection Fraction	1.05	1.01	1.10	0.018
Combination PA–HS				0.038
PA > 5.15–HS > 30.7	1.00 (reference)			–
PA > 5.15–HS < 30.7	2.19	0.90	5.32	0.084
PA < 5.15–HS > 30.7	2.97	0.85	10.46	0.090
PA < 5.15–HS < 30.7	4.02	1.53	10.56	0.005

Α multivariate regression model for the prediction of ICU stay using PA and HS as continuous variables was also performed and is presented in Additional file 1: Table S2. According to that analysis, increased HS was independently associated with decreased ICU LOS (aOR = 0.95, 95% CI: 0.90–0.99).

In order to observe the relationship between the PA–HS combination and the standard EuroSCORE II risk index, an analysis of variance (ANOVA) was performed, showing a statistically significant difference in EuroSCORE II values among individuals with PA < 5.15–HS < 25.5 compared to those with PA > 5.15–HS > 25.5 in mortality (Table 9).

**Table 9:** EUROSCORE II relationship with PA–HS combination index

Combination PA–HS	Combination PA–HS	Sig
below 5.15–below 25.5	below 5.15–above 25.5	1.000
	above 5.15–below 25.5	0.558
	above 5.15–above 25.5	** < 0.001**
below 5.15–above 25.5	above 5.15–below 25.5	1.000
	above 5.15–above 25.5	0.119
above 5.15–below 5.5	above 5.15–above 25.5	0.932

Dependent variable: EUROSCORE II (%)

Statistical tests of collinearity were also performed for each one of the multivariate analyses to investigate if there is any source of collinearity which could decrease the value of the independent effect (Additional file 1: Table S3). No significant source of collinearity was identified since every calculated Tolerance was greater than 0.2 and the calculated variance inflation factors (VIFs) were relatively low.

## Discussion

The results of this prospective observational study suggest the potential prognostic value of the combined preoperative PA–HS measurement as a new biomarker for predicting one-year mortality in cardiac patients undergoing selective cardiac surgery. According to our analyses, patients having a combination of low PA and HS values were 5 times more likely to die and 4 times more likely to remain in the ICU for more than one postoperative day, thus increasing postoperative morbidity and the likelihood of complications. Furthermore, the EuroSCORE II index, an internationally established risk index for death after cardiac surgery, was associated with the combination of PA–HS. To our knowledge, this is the first study examining whether the combination of PA and HS values is associated with one-year mortality rates and early morbidity in patients undergoing cardiac surgery, and could thereby enhance their pre-operative risk stratification.

From the literature review, low PA is associated with nutritional risk, increased morbidity and mortality in immunocompromised patients or patients with chronic kidney disease [8]. It has been particularly associated with poor functional status and worse prognosis in cancer patients [8]. PA is often lower than normal in diseased individuals, since infection, systemic inflammation or specific parameters of a disease may cause cell destruction and consequent reduction in PA [8, 9]. PA is positively correlated with total body protein, muscle mass, offering a qualitative dynamic aspect of the body's functional state [10]. Moreover, low PA values have been associated with weakness, vulnerability (frailty) and mortality, regardless of age and other comorbidities [11, 12].

According to the BICS (Bioimpedance in Cardiac Surgery) study enrolling 277 patients undergoing major cardiac surgery in Canada, PA can independently predict early and midterm mortality after major cardiac surgery [6]. A PA cutoff of < 4.5° had the highest predictive value for 1-year mortality, and every 1° decrease in PA conferred an almost threefold higher risk of mortality. Our analysis yielded a cut-off point of PA = 5.15° for the prediction of increased mortality and ICU stay. Additionally, PA has been suggested as a dynamic marker with the potential to respond to targeted interventions aiming to restore adequate nutritional status, increase physical activity, and optimize fluid status [6].

The measurement of HS is the most commonly used indicator for muscle function in several clinical conditions, as it is considered a strong indicator of functional capacity of the muscles as well as an indicative point of a patient’s nutritional status. The correlation between nutritional status and HS is well documented [13, 14]. Previous studies have also shown that HS was correlated with the severity of the disease, with aging and mortality in elderly individuals [15, 16]. Particularly in cardiac surgery, HS has been well-recognized as a preoperative risk assessment tool since weak HS has been associated with 1-year and 30-day mortality, heart failure, kidney disease, malnutrition, and various frailty scales [17–19]. Hence, our study concurs with the growing body of literature regarding the poor outcomes of cardiac surgery patients with low preoperative PA and HS, and adds that their combined assessment might be an option to consider as a risk stratification tool.

Nevertheless, our study is subject to several limitations. Τhe small sample size, the limited follow-up and the monocentric study design restrict the generalizability of our findings. Secondly, we could not precisely record the dates of patients’ deaths and, therefore, survival analyses could not be performed. Furthermore, major adverse cardiovascular events and re-admissions were not documented in our database. Additionally, PA was not standardized for its direct determinants: age, gender and BMI, which could affect our results; however, we have adjusted our multivariate analyses for age and gender, while BMI was not univariately associated with either mortality or ICU LOS. Nevertheless, PA standardization prior to statistical analyses might be considered in future studies [20, 21]. Moreover, the characterization of prolonged ICU-stay as staying in ICU for more than 1 day was based on relevant studies [22–25], but not analyzing it as a continuous variable might cause potential misinterpretation of our results. However, PA and HS have not been sufficiently studied in the specific patient population; hence, our results seem to be promising for their utilization and could trigger future studies to combine these biomarkers and associate them with postoperative prognosis. Thereby, we could ultimately achieve a more detailed holistic risk stratification of patients undergoing cardiac surgery and possibly direct them towards other alternative treatments such as angioplasty, valvular replacement or optimal palliative care.

## Conclusions

In this prospective observational study, the combination of low preoperative PA and HS values was significantly associated with higher risk of all-cause mortality at 12 months and prolonged ICU stay. Hence, these clinical biomarkers could serve as prognostic tools for assessing adverse clinical course after cardiac surgery procedures. Larger studies and randomized-controlled trials are needed to confirm these results.

## Supplementary Information

**Additional file 1:** Supplementary tables S1, S2, and S3.

## Data Availability

The dataset supporting the conclusions of this article is available from Georgios Tagarakis (e-mail: gtagarakis@gmail.com) upon reasonable request and with permission of AHEPA University Hospital.

## References

[1] Al-Ebrahim EK, Madani TA, Al-Ebrahim KE (2022). Future of cardiac surgery, introducing the interventional surgeon. J Cardiac Surg.

[2] Hote M (2018). Cardiac surgery risk scoring systems: In quest for the best. Heart Asia.

[3] Serra R, Jiritano F, Bracale UM (2021). Novel biomarkers in cardiovascular surgery. Biomark Med.

[4] Richter D, Guasti L, Walker D (2022). Frailty in cardiology: definition, assessment and clinical implications for general cardiology. A consensus document of the Council for Cardiology Practice (CCP), Association for Acute Cardio Vascular Care (ACVC), Association of Cardiovascular Nursing and. Eur J Prev Cardiol.

[5] Minto G, Biccard B (2014). Assessment of the high-risk perioperative patient. Contin Educ Anaesth Crit Care Pain.

[6] Mullie L, Obrand A, Bendayan M (2018). Phase angle as a biomarker for frailty and postoperative mortality: the BICS study. J Am Heart Assoc.

[7] Sultan P, Hamilton MA, Ackland GL (2012). Preoperative muscle weakness as defined by handgrip strength and postoperative outcomes: a systematic review. BMC Anesthesiol.

[8] Arab A, Karimi E, Vingrys K, Shirani F (2021). Is phase angle a valuable prognostic tool in cancer patients’ survival? a systematic review and meta-analysis of available literature. Clin Nutr.

[9] Schwenk A, Beisenherz A, Römer K, Kremer G, Salzberger B, Elia M (2000). Phase angle from bioelectrical impedance analysis remains an independent predictive marker in HIV-infected patients in the era of highly active antiretroviral treatment. Am J Clin Nutr.

[10] Norman K, Stobäus N, Pirlich M, Bosy-Westphal A (2012). Bioelectrical phase angle and impedance vector analysis–clinical relevance and applicability of impedance parameters. Clin Nutr.

[11] Alves FD, Souza GC, Clausell N, Biolo A (2016). Prognostic role of phase angle in hospitalized patients with acute decompensated heart failure. Clin Nutr.

[12] Wilhelm-Leen ER, Hall YN, Horwitz RI, Chertow GM (2014). Phase angle, frailty and mortality in older adults. J Gen Intern Med.

[13] Pratama IK, Setiati S (2018). Correlation between hand grip strength and functional mobility in elderly patients. J Phys Conf Ser.

[14] Norman K, Stobäus N, Gonzalez MC, Schulzke J-D, Pirlich M (2011). Hand grip strength: outcome predictor and marker of nutritional status. Clin Nutr.

[15] Kilgour RD, Vigano A, Trutschnigg B, Lucar E, Borod M, Morais JA (2013). Handgrip strength predicts survival and is associated with markers of clinical and functional outcomes in advanced cancer patients. Support Care Cancer Off J Multinatl Assoc Support Care Cancer.

[16] Ling CHY, Taekema D, de Craen AJM, Gussekloo J, Westendorp RGJ, Maier AB (2010). Handgrip strength and mortality in the oldest old population: the Leiden 85-plus study. C Can Med Assoc J J l’Assoc Med Can.

[17] Fountotos R, Munir H, Goldfarb M (2021). Prognostic value of handgrip strength in older adults undergoing cardiac surgery. Can J Cardiol.

[18] Perry IS, Pinto LC, da Silva TK, Vieira SRR, Souza GC (2019). Handgrip strength in preoperative elective cardiac surgery patients and association with body composition and surgical risk. Nutr Clin Pract Off Publ Am Soc Parenter Enter Nutr.

[19] Fu L, Zhang Y, Shao B (2019). Perioperative poor grip strength recovery is associated with 30-day complication rate after cardiac surgery discharge in middle-aged and older adults - a prospective observational study. BMC Cardiovasc Disord.

[20] Garlini LM, Alves FD, Ceretta LB, Perry IS, Souza GC, Clausell NO (2019). Phase angle and mortality: a systematic review. Eur J Clin Nutr.

[21] de Borba EL, Ceolin J, Ziegelmann PK (2022). Phase angle of bioimpedance at 50 kHz is associated with cardiovascular diseases: systematic review and meta-analysis. Eur J Clin Nutr.

[22] Almashrafi A, Elmontsri M, Aylin P (2016). Systematic review of factors influencing length of stay in ICU after adult cardiac surgery. BMC Health Serv Res.

[23] Janssen DPB, Noyez L, Wouters C, Brouwer RMHJ (2004). Preoperative prediction of prolonged stay in the intensive care unit for coronary bypass surgery. Eur J Cardio-Thoracic Surg Off J Eur Assoc Cardio-Thoracic Surg.

[24] Atoui R, Ma F, Langlois Y, Morin J-F (2008). Risk factors for prolonged stay in the intensive care unit and on the ward after cardiac surgery. J Card Surg.

[25] Nilsson J, Algotsson L, Höglund P, Lührs C, Brandt J (2004). EuroSCORE predicts intensive care unit stay and costs of open heart surgery. Ann Thorac Surg.

