# Supplementary material for: Prognostic value of combined preoperative phase angle and handgrip strength in cardiac surgery
Source: J Cardiothorac Surg. 2022 Sep 3;17:227. doi: 10.1186/s13019-022-01970-z (PMC9440499; doi:10.1186/s13019-022-01970-z)
Supplement: Supplementary file 1 — Additional file 1: Supplementary tables S1, S2, and S3. [file 13019_2022_1970_MOESM1_ESM.docx]

**Prognostic value of combined preoperative phase angle and handgrip strength in cardiac surgery**

**This appendix has been provided by the authors to give readers additional information about their work.**

**Contents**

| Table S1. | Multivariate regression model for the prediction of 1-year mortality using Phase Angle and Handgrip Strength as continuous variables |
| --- | --- |
| Table S2. | Multivariate regression model for the prediction of ICU stay using Phase Angle and Handgrip Strength as continuous variables |
| Table S3. | Collinearity statistics among the variables included into the multivariate regression models |

**Table S1.** Multivariate regression model for the prediction of 1-year mortality using Phase Angle and Handgrip Strength as continuous variables

| Variable: | aOR (95% CI) | p-value |
| --- | --- | --- |
|  |  |  |
| Female gender | 0.19 (0.05-0.74) | **0.017** |
| Age | 0.99 (0.94-1.05) | 0.936 |
| Diabetes II | 0.99 (0.37-2.59) | 0.988 |
| Chronic kidney disease | 0.56 (0.19-1.63) | 0.295 |
| Euroscore ΙΙ | 1.57 (1.44-2.16) | **0.005** |
| Ejection fraction | 0.97 (0.91-1.02) | 0.305 |
| Phase Angle | 0.74 (0.46-1.19) | 0.220 |
| Handgrip strength | 0.90 (0.84-0.96) | **0.003** |

**Table S2.** Multivariate regression model for the prediction of ICU stay using Phase Angle and Handgrip Strength as continuous variables

| Variable: | aOR (95% CI) | p-value |
| --- | --- | --- |
|  |  |  |
| Female gender | 0.33 (0.13-0.87) | **0.025** |
| Age | 1.03 (0.99-1.07) | 0.100 |
| Diabetes II | 1.66 (0.84-3.29) | 0.141 |
| Chronic kidney disease | 0.59 (0.26-1.34) | 0.213 |
| Euroscore ΙΙ | 1.80 (1.33-2.45) | **0.000** |
| Ejection fraction | 1.04 (1.00-1.08) | 0.047 |
| Phase Angle | 0.96 (0.76-1.21) | 0.731 |
| Handgrip strength | 0.95 (0.90-0.99) | **0.041** |

Table S3: Collinearity statistics among the variables included into the multivariate regression models.

|  | Tolerance | VIF |
| --- | --- | --- |
| Female Gender | 0.72 | 1.39 |
| Age | 0.64 | 1.56 |
| Diabetes ΙΙ | 0.55 | 1.82 |
| Chronic Kidney Disease | 0.61 | 1.64 |
| EuroscoreΙΙ | 0.30 | 3.33 |
| Ejection Fraction | 0.43 | 2.33 |
| Combination PA-HS | 0.38 | 2.63 |
